# Clinical Performance of the RealTi*m*e High Risk HPV Assay on Self-Collected Vaginal Samples within the VALHUDES Framework

**DOI:** 10.1128/spectrum.01631-22

**Published:** 2022-09-01

**Authors:** Ardashel Latsuzbaia, Davy Vanden Broeck, Severien Van Keer, Steven Weyers, Wiebren A. A. Tjalma, Jean Doyen, Gilbert Donders, Philippe De Sutter, Alex Vorsters, Eliana Peeters, Marc Arbyn

**Affiliations:** a Unit of Cancer Epidemiology, Belgian Cancer Centre, Sciensano, Brussels, Belgium; b Laboratory of Molecular Pathology, AML Sonic Healthcare, Antwerp, Belgium; c National Reference Centre for HPV, Brussels, Belgium; d Applied Molecular Biology Research Group (AMBIOR), Laboratory for Cell Biology and Histology, University of Antwerp, Antwerp, Belgium; e International Centre for Reproductive Health, Ghent University, Ghent, Belgium; f Centre for the Evaluation of Vaccination (CEV), Vaccine and Infectious Disease Institute (VAXINFECTIO), Faculty of Medicine and Health Sciences, University of Antwerp, Wilrijk (Antwerp), Belgium; g Department of Obstetrics and Gynaecology, Ghent University Hospital, Ghent, Belgium; h Multidisciplinary Breast Clinic, Unit Gynaecologic Oncology, Department of Obstetrics and Gynaecology, Antwerp University Hospital (UZA), Edegem, Belgium; i Molecular Imaging, Pathology, Radiotherapy, Oncology (MIPRO), Faculty of Medicine and Health Sciences, University of Antwerp, Antwerp, Belgium; j Department Gynaecology-Obstetrics, University Hospital Liège, Liège, Belgium; k Department of Obstetrics and Gynaecology of the General Regional Hospital Heilig Hart, Tienen, Belgium; l Femicare vzw, Clinical Research for Women, Tienen, Belgium; m Department of Obstetrics and Gynaecology, University Hospital Antwerp, Antwerp, Belgium; n Department Gynaecology-Oncology, UZ Brussels-VUB, Brussels, Belgium; o Department of Human Structure and Repair, Faculty of Medicine and Health Sciences, University Ghent, Ghent, Belgium; University of Arizona/Banner Health

**Keywords:** Abbott RealTi*m*e High Risk HPV assay, HPV, VALHUDES, cervical cancer screening, diagnostic test accuracy, self-sampling

## Abstract

The VALHUDES framework (NCT03064087) was established to evaluate the clinical accuracy of HPV testing on self-samples compared with HPV testing on matched clinician-taken cervical samples. Women referred to colposcopy due to previous cervical abnormalities were recruited at five Belgian colposcopy centers. A total of 486 pairs of matched cervical samples and vaginal self-samples were included in the analysis (228 collected with Evalyn Brush and 258 with Qvintip). The dry vaginal brushes were transferred into 20 mL ThinPrep PreservCyt solution. All specimens were tested with the Abbott RealTi*m*e High Risk HPV assay (Abbott RT). Testing on vaginal and cervical specimens was considered the index and comparator tests, respectively, and colposcopy and histology as the reference standard. The clinical sensitivity for CIN2+ of Abbott RT (cutoff ≤32 cycle number [CN]) on vaginal self-samples (Evalyn Brush and Qvintip combined) was 8% lower than on the cervical clinician-collected samples (ratio = 0.92 [95% CI, 0.87 to 0.98]), while the specificity was similar (ratio = 1.04 [95% CI, 0.97 to 1.12]). Sensitivity (ratio = 0.95 [95% CI, 0.89 to 1.02]) and specificity (ratio = 1.11 [95% CI, 0.995 to 1.23]) on Evalyn Brush samples was similar to cervical, while on Qvintip samples, the sensitivity was 12% lower than cervical samples (ratio = 0.88 [95% CI, 0.78 to 0.998]) with similar specificity (0.99 [95% CI, 0.90 to 1.10]). Exploratory cutoff optimization (cutoff ≤35 CN) resulted in an improvement of the relative sensitivity (self-sampling versus clinician sampling: ratio = 0.96 [95% CI, 0.91 to 1.02]) but yielded a loss in relative specificity (ratio = 0.92 [0.85 to 1.00]). The clinical accuracy of Abbott RT differed from the self-sampling device. However, after cutoff optimization, the sensitivity on self-samples taken with either of two vaginal brushes became similar to clinician-collected samples.

**IMPORTANCE** Self-samples are becoming a crucial part of HPV-based cervical cancer screening programs to reach nonattendee women and increase screening coverage. Therefore, the VALHUDES framework was established to validate and evaluate HPV tests and devices on self-samples. Here, in the present manuscript, we evaluated the accuracy of the RealTi*m*e High Risk HPV assay (Abbott RT) on two different vaginal devices to detect cervical intraepithelial neoplasia grade two or higher (CIN2+). The study results demonstrated that the Abbott RT assay is similarly accurate on vaginal self-samples as on matched clinician-taken cervical samples after adjusting cutoff values. Moreover, we observed that some vaginal devices perform better than others in CIN2+ detection. We also underline the necessity of standardization and validation of general workflow and sample handling procedures for vaginal self-samples.

## INTRODUCTION

Invasive cervical cancer (ICC) caused by a persistent high-risk human papillomavirus (hrHPV) infection is largely preventable with screening and vaccination programs in place (1, 2). The introduction of cervical cancer screening programs has led to a significant reduction in the incidence and mortality of ICC in Europe and worldwide (1). Nevertheless, a considerable proportion of women are rarely or never screened and therefore remain at the highest risk of developing ICC (3, 4). Under-screened women can be reached out by offering vaginal or urine self-sampling devices for HPV testing, which usually is more effective to trigger response than a conventional invitation to contact a clinician for taking a Pap smear (5).

Recent studies have shown that HPV-based cervical cancer screening is more effective and provides longer protection against ICC compared to cytology (6, 7). The new evidence guided several countries worldwide to introduce HPV-based primary cervical cancer screening (8–10). A key advantage of HPV-based screening is that a self-sample can be easily collected at home and then shipped and tested in the laboratory. Thus, several countries are executing pilot studies or have introduced self-sampling as an alternative to reach out nonattendees (10, 11). A meta-analysis by Arbyn et al. (5) has reported that HPV testing with PCR (PCR)-based assays on vaginal self-samples is similarly sensitive compared to clinician-taken cervical samples to detect cervical intraepithelial neoplasia grade 2 or higher (CIN2+). The sensitivity of HPV tests on both vaginal and urine self-samples varies considerably across the general mode of HPV detection procedure (5, 12). Several hundreds of HPV tests are currently commercially available on the market, only a few of which have been validated for cervical cancer screening following international consensus guidelines on cervical samples (13, 14), but not on vaginal nor urine samples. However, robust validation principles are only established for HPV testing on clinician-based samples (15). Therefore, we developed a standard protocol for validation of HPV assays and collection devices for HPV testing on vaginal and urine self-samples (VALHUDES) (16). The first report from VALHUDES concluded that the clinical accuracy of the Abbott RealTi*m*e High Risk HPV assay (Abbott RT) (Abbott GmbH, Wiesbaden, Germany) on first-void urine collected with Colli-Pee device (Novosanis, Wijnegem, Belgium) was similarly performant to detect CIN2+ compared to cervical samples (17).

This study aimed to evaluate the analytical and clinical performance of this test on vaginal self-samples collected with Evalyn Brush or Qvintip against clinician-taken cervical samples.

## RESULTS

### Study characteristics.

**FIG 1:**
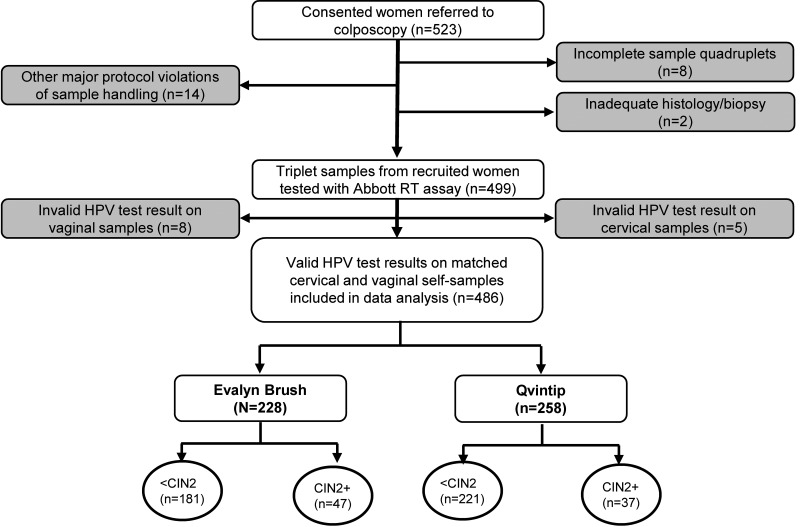
Flow chart of samples included in the VALHUDES trial tested with the Abbott RealTi*m*e High Risk HPV (Abbott RT) assay. Gray boxes represent excluded samples. Detailed exclusions are reported in the Supplemental Material.

A total of 486 matched vaginal and clinician-taken cervical samples were included in the study with the valid HPV test result. Five sample pairs were excluded due to invalid HPV test results on cervical and eight others on vaginal self-samples, whereas 24 other pairs were excluded for protocol violations described elsewhere (Fig. 1 and Supplemental File 1) (17). Thirteen cervical and 24 vaginal (14 Evalyn Brush, 10 Qvintip) samples were retested due to initial test failure of which 8 cervical and 16 vaginal (9 Evalyn Brush, 7 Qvintip) samples had a valid second test result (18).

Details on the study population have been previously reported (17), in the brief median age of participants was 40 years (range 19 to 72, IQR 31 to 50). Biopsy was taken for 59% (288/486), while no biopsy specimen was required for 41% (198/486). Of 288 study subjects with biopsy specimen outcomes, 29% (84/288) had CIN2+, 15% (43/288) had CIN3+ and 71% (204/288) had CIN1 or no CIN. The median age was significantly lower in women with CIN2+ (35 years, IQR 29 to 44) compared to women with <CIN2 (41 years, IQR 32 to 50) (Mann-Whitney test, *P* = 0.004). Eighty percent (390/486) of study participants were within the recommended target age for HPV-based cervical cancer screening (age ≥30 years old, Table S1 in Supplemental File 1). Details of the study population by age group are shown in Table S1 in Supplemental File 1.

### Clinical accuracy.

The clinical sensitivity of HPV testing on cervical and vaginal (Evalyn and Qvintip combined) samples was 92.9% (95% CI, 85.1 to 97.3%) and 85.7% (95% CI, 76.4 to 92.4%) for CIN2+ and 97.7% (95% CI, 87.7 to 99.9%) and 88.4% (95% CI, 74.9 to 96.1%) for CIN3+, respectively. Clinical sensitivity for CIN2+ on samples collected with Evalyn Brush was slightly higher than on samples collected with Qvintip (89.4% versus 81.1% [ratio = 0.91, 95%CI 0.75 to 1.09]) (Table 1).

**TABLE 1:** Clinical sensitivity and specificity of the RealTi*m*e High Risk HPV assay on vaginal self-samples and cervical clinician-collected samples

CN cutoff	N^*a*^	CIN2+ sensitivity (95% CI)	N	CIN3+ sensitivity (95% CI)	N	<CIN2 specificity (95% CI)
Women ≥30 yrs old (*N* = 390), CN ≤32^*b*^						
Cervical	59/61	91.8 (81.9–97.3)	32/33	97.0 (84.2–99.9)	164/329	49.8 (44.3–55.4)
Vaginal (E + Q)	51/61	83.6 (71.9–81.8)	29/33	87.9 (71.8–96.6)	172/329	52.3 (46.7–57.8)
Evalyn	29/32	90.6 (75.0–98.0)	15/17	88.2 (63.6–98.5)	82/155	52.9 (44.7–61.0)
Qvintip	22/29	75.9 (56.5–89.7)	14/16	87.5 (61.7–98.4)	90/174	51.7 (44.0–59.4)
Total study population (*N* = 486), CN ≤32^*b*^						
Cervical	78/84	92.9 (85.1–97.3)	42/43	97.7 (87.7–99.9)	194/402	48.3 (43.3–53.3)
Vaginal (E + Q)	72/84	85.7 (76.4–92.4)	38/43	88.4 (74.9–96.1)	202/402	50.2 (45.3–55.2)
Evalyn	42/47	89.4 (76.9–96.5)	20/23	87.0 (66.4–97.2)	94/181	51.9 (44.3–59.4)
Qvintip	30/37	81.1 (64.8–92.0)	18/20	90.0 (68.3–98.8)	108/221	48.9 (42.1–55.6)
Women ≥30 yrs old (*N* = 390), CN ≤35^*c*^						
Vaginal (E + Q)	53/61	86.9 (75.8–94.2)	29/33	87.9 (71.8–96.6)	155/329	47.1(41.6–52.7)
Evalyn	29/32	90.6 (75.0–98.0)	15/17	88.2 (63.6–98.5)	76/155	49.0 (40.9–57.2)
Qvintip	24/29	82.8 (64.2–94.1)	14/16	87.5 (61.7–98.4)	76/155	45.4 (37.9–53.1)
Total study population (*N* = 486), CN ≤35^*c*^						
Vaginal (E + Q)	75/84	89.3 (80.6–95.0)	39/43	90.7 (77.9–97.4)	179/402	44.5 (39.6–49.5)
Evalyn	43/47	91.5 (79.6–97.6)	21/23	91.3 (72.0–98.9)	86/181	47.5 (40.1–55.1)
Qvintip	32/37	86.5 (71.2–95.5)	18/20	90.0 (68.3–98.8)	93/221	42.1 (35.4–48.9)

a CI, 95% confidence interval; CIN, cervical intraepithelial neoplasia; E + Q, samples collected with Evalyn Brush (E) and Qvintip (Q) combined; N, number.

b Cutoff **≤**32 cycle numbers (CN) as predefined by the manufacturer.

c Exploratory cutoff ≤35 CNs for vaginal samples.

The relative sensitivity values are shown in Table 2. Clinical sensitivity for CIN2+ on vaginal self-samples (Evalyn and Qvintip combined) was 8% lower than on cervical samples (ratio = 0.92 [95% CI, 0.87 to 0.98]) and specificity for <CIN2 was similar (ratio = 1.04 [95% CI, 0.97 to 1.12]). Clinical sensitivity for CIN2+ on samples collected with Evalyn Brush was similar to cervical (ratio = 0.95 [95% CI, 0.89 to 1.02]) and specificity was higher than on cervical samples (ratio = 1.11 [95% CI, 0.995 to 1.23]). Whereas, on samples collected with Qvintip, sensitivity was 12% lower (ratio = 0.88 [95% CI, 0.78 to 0.998]) with similar specificity (ratio = 0.99 [95% CI, 0.90 to 1.10]) (Table 2).

**TABLE 2:** Relative sensitivity and specificity of the RealTi*m*e High Risk HPV assay on vaginal self-samples versus cervical clinician-collected samples

CN cutoff	CIN2+ sensitivity (95% CI)^*a*^	CIN3+ sensitivity (95% CI)	<CIN2 specificity (95% CI)
Women ≥30 yrs old (N = 390), CN ≤ 32^*b*^			
Vaginal (E + Q)	0.91 (0.84–0.99)	0.91 (0.81–1.01)	1.05 (0.97–1.14)
Evalyn	0.97 (0.90–1.03)	0.94 (0.83–1.06)	1.11 (0.996–1.23)
Qvintip	0.85 (0.72–0.997)	0.88 (0.73–1.05)	1.00 (0.89–1.12)
Total study population (*N* = 486), CN ≤ 32^*b*^			
Vaginal (E + Q)	0.92 (0.87–0.98)	0.90 (0.82–0.998)	1.04 (0.97–1.12)
Evalyn	0.95 (0.89–1.02)	0.91 (0.80–1.04)	1.11 (1.00–1.30)
Qvintip	0.88 (0.78–0.998)	0.90 (0.78–1.04)	0.99 (0.90–1.10)
Women ≥ 30 yrs old (*N* = 390), CN ≤ 35^*c*^			
Vaginal (E + Q)	0.95 (0.87–1.03)	0.91 (0.81–1.01)	0.95 (0.87–1.03)
Evalyn	0.97 (0.90–1.03)	0.94 (0.83–1.06)	1.03 (0.93–1.14)
Qvintip	0.92 (0.79–1.08)	0.88 (0.73–1.05)	0.88 (0.77–0.99)
Total study population (*N* = 486), CN ≤ 35^*c*^
Vaginal (E + Q)	0.91 (0.84–0.99)	0.91 (0.81–1.01)	1.05 (0.97–1.14)
Evalyn	0.97 (0.90–1.03)	0.94 (0.83–1.06)	1.11 (0.996–1.23)
Qvintip	0.85 (0.72–0.997)	0.88 (0.73–1.05)	1.00 (0.89–1.12)

a CI, 95% confidence interval; CIN, cervical intraepithelial neoplasia; E+Q, samples collected with Evalyn Brush (E) and Qvintip (Q) combined; N, number.

b Cutoff **≤** 32 cycle numbers (CN) as predefined by the manufacturer.

c Exploratory cutoff ≤35 CNs for vaginal samples.

When the cutoff for HPV positivity on vaginal samples was defined at CN ≤35, three additional CIN2+ and one additional CIN3+ cases were detected improving the clinical sensitivity to 89.3% (95% CI, 80.6 to 95.0%) and 90.7% (95% CI, 77.9 to 97.4%), respectively. Increasing the CN threshold to ≤35 resulted in 23 additional false-positive cases with a decrease in specificity to 44.5% (95% CI, 39.6 to 49.5%). The change of test cutoff resulted in a similar sensitivity compared to cervical specimens for vaginal samples combined (Evalyn Brush + Qvintip), but lower specificity for vaginal specimens overall and the Qvintip brushes (Table 2).

When restricting the analysis to women 30 years and older similar relative and clinical accuracy was observed for both devices compared to the total population (Table 1 and 2). The clinical sensitivity for CIN2+ in ≥30 years old women on cervical samples (91.8% [95%CI 81.9 to 97.3%]) was higher than on vaginal samples (83.6% [95% CI, 71.9 to 81.8%]) with corresponding relative sensitivity excluding unity (ratio = 0.92 [95% CI, 0.87 to 0.98]), whereas clinical specificity for <CIN2 on cervical (49.8% [95% CI, 44.3 to 55.4%]) and vaginal samples (52.3% [95% CI, 46.7 to 57.8%]) was similar (ratio = 1.05 [95% CI, 0.97 to 1.14]) (Table 1 and 2).

### Analytical performance.

Overall, hrHPV positivity (cutoff CN ≤32), was 59% (286/486) on cervical and 56% (272/486) on vaginal samples, while 51% (249/486) of cases were hrHPV positive in both specimens.

Good and excellent agreement was observed for overall hrHPV positivity, HPV16, HPV18, and other hrHPV (with cutoff ≤32 and ≤35 CN) between vaginal samples combined (Evalyn Brush + Qvintip) as well as for each device separately with Kappa values ranging from 0.65 to 1.00 (Table 3 and Table S2 in Supplemental File 1). Cutoff optimization resulted in 12 additional samples concordantly identified as hrHPV positive on both sample types, whereas 14 samples were reclassified as hrHPV positive on vaginal samples but not on cervical (Table 3 and Table S2A in Supplemental File 1).

**TABLE 3:** Type-specific test concordance and agreement between vaginal and cervical samples

N^*a*^	HPV type^*b*^	+/+^*c*^	+/−	−/+	−/−	Concordance (%)	Kappa (95% CI)^*d*^
Vaginal (Evalyn + Qvintip) and cervical^*e*^							
Total population (N = 486)	HrHPV	249	37	23	177	87.65	0.748 (0.688–0.807)
	HPV16	63	4	8	411	97.53	0.899 (0.842–0.955)
	HPV18	16	5	2	463	98.56	0.813 (0.678–0.948)
	Other hrHPV	196	40	25	225	86.63	0.732 (0.671–0.792)
CIN3^+^ (N = 43)	HrHPV	38	4	0	1	90.70	0.306 (−0.160–0.773)
	HPV16	24	1	0	18	97.67	0.953 (0.861–1.000)
	HPV18	2	0	0	41	100	1.000 (1.000–1.000)
	Other hrHPV	18	5	2	18	83.72	0.676 (0.458–0.894)
CIN2^+^ (N = 84)	HrHPV	72	6	0	6	92.86	0.632 (0.367–0.896)
	HPV16	36	2	1	45	96.43	0. 928 (0.847–1.000)
	HPV18	3	0	0	81	100	1.000 (1.000–1.000)
	Other hrHPV	45	7	3	29	88.10	0.754 (0.611–0.896)
<CIN2 (N = 402)	HrHPV	177	31	23	171	86.57	0.731 (0.665–0.798)
	HPV16	27	2	7	366	97.76	0.845 (0.746–0.944)
	HPV18	13	5	2	382	98.26	0.779 (0.620–0.938)
	Other hrHPV	151	33	22	196	86.32	0.723 (0.655–0.791)
Evalyn Brush and cervical						
Total population (N = 228)	HrHPV	122	18	7	81	89.04	0.774 (0.691–0.857)
	HPV16	38	2	1	187	98.68	0.954 (0.902–1.000)
	HPV18	12	4	1	211	97.81	0.816 (0.659–0.973)
	Other hrHPV	86	20	10	228	86.84	0.734 (0.646–0.822)
CIN3^+^ (N = 23)	HrHPV	20	2	0	1	91.30	0.465 (−0.133–1.000)
	HPV16	17	1	0	5	95.65	0.881 (0.654–1.000)
	HPV18	0	0	0	23	NA	N/A
	Other hrHPV	5	3	1	14	82.61	0.593 (0.242–0.944)
CIN2^+^ (N = 47)	HrHPV	42	2	0	3	95.74	0.728 (0.374–1.000)
	HPV16	26	2	0	19	95.74	0.913 (0.796–1.000)
	HPV18	1	0	0	46	100	1.000 (1.000–1.000)
	Other hrHPV	21	3	2	21	89.36	0.787 (0.611–0.963)
<CIN2 (N = 181)	HrHPV	80	16	7	78	87.29	0.746 (0.650–0.843)
	HPV16	12	0	1	168	99.45	0.957 (0.873–1.000)
	HPV18	11	4	1	165	97.24	0.800 (0.630–0.970)
	Other hrHPV	65	17	8	91	86.19	0.719 (0.617–0.820)
Qvintip and cervical							
Total population (N = 258)	HrHPV	127	19	16	96	86.43	0.725 (0.640–0.809)
	HPV16	25	2	7	224	96.51	0.828 (0.719–0.937)
	HPV18	4	1	1	252	99.22	0.796 (0.520–1.000)
	Other hrHPV	110	20	15	113	86.43	0.729 (0.645–0.812)
CIN3^+^ (N = 20)	HrHPV	18	2	0	0	90.00	NA
	HPV16	7	0	0	13	100	1.000 (1.000–1.000)
	HPV18	2	0	0	18	100	1.000 (1.000–1.000)
	Other hrHPV	13	2	1	4	85.00	0.625 (0.243–1.000)
CIN2^+^ (N = 37)	HrHPV	30	4	0	3	89.19	0.549 (0.176–0.921)
	HPV16	10	0	1	26	97.30	0.934 (0.805–1.000)
	HPV18	2	0	0	35	100	1.000 (1.000–1.000)
	Other hrHPV	24	4	1	8	86.49	0.670 (0.409–0.932)
<CIN2 (N = 221)	HrHPV	97	15	16	93	85.97	0.719 (0.628–0.811)
	HPV16	15	2	6	198	96.38	0.770 (0.617–0.923)
	HPV18	2	1	1	217	99.10	0.662 (0.222–1.000)
	Other hrHPV	86	16	14	105	86.43	0.727 (0.636–0.817)

a N, number; CI, 95% confidence interval; CIN, cervical intraepithelial neoplasia; NA, not applicable.

b Cutoff **≤**32 cycle numbers (CN) as predefined by the manufacturer.

c +/+ positive on vaginal and cervical samples, +/− positive only on cervical samples, −/+ positive only on vaginal samples, −/− negative on both sample types.

d Kappa concordance between the vaginal and cervical samples was 0 to 0.2, poor; 0.21 to 0.4, fair; 0.41 to 0.6, moderate; 0.61 to 0.8, good; 0.81 to 1.0, excellent.

e Concordance between vaginal (CN ≤35) and cervical samples (CN ≤32) is presented in Table S2A to C in Supplemental File 1.

**FIG 2:**
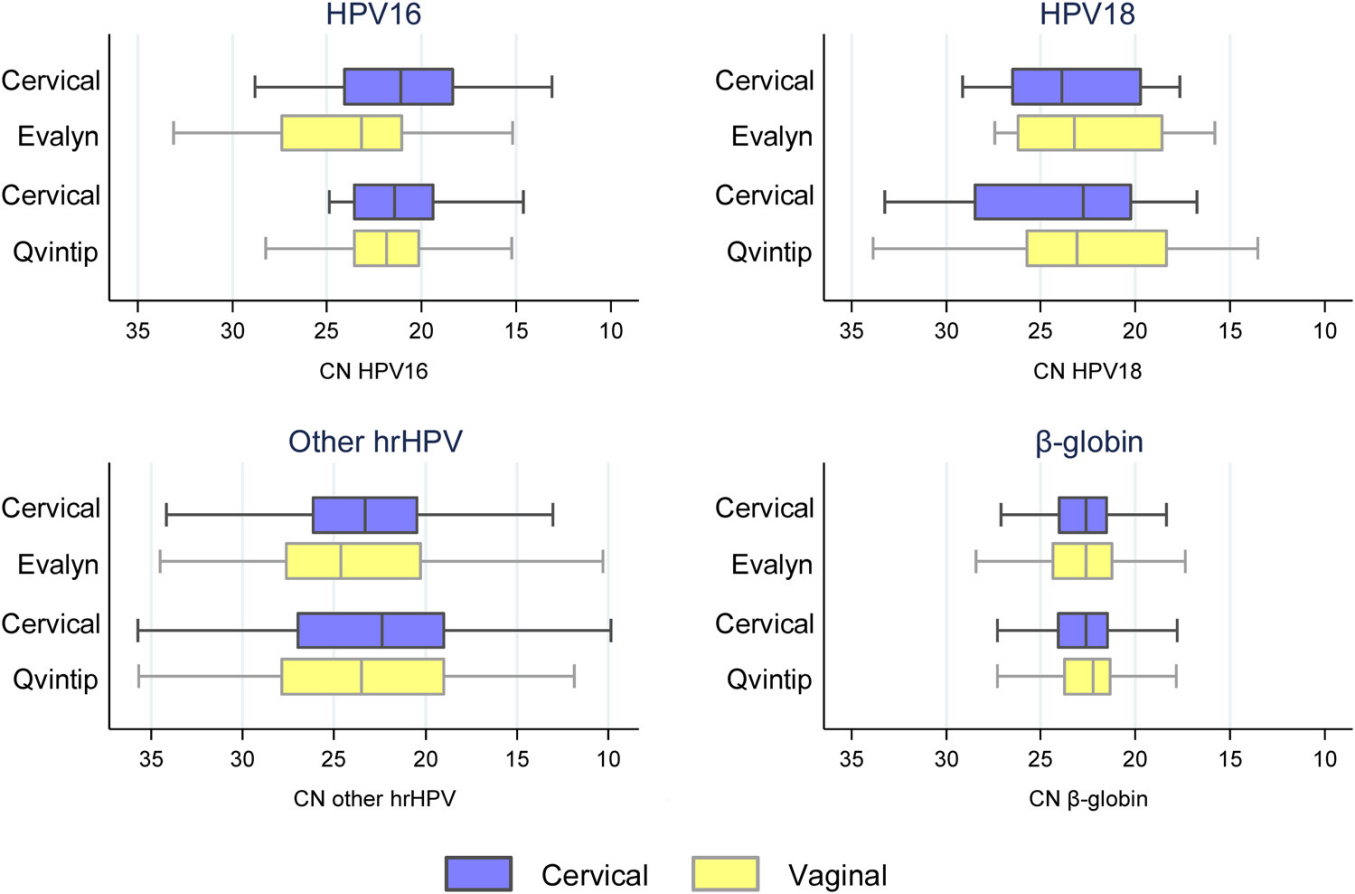
RealTi*m*e High Risk HPV cycle number (CN) values between cervical and vaginal samples were collected with Evalyn Brush or Qvintip. Boxplots indicate median CN values, interquartile ranges, and extreme values (whiskers) for HPV16, 18, other hrHPV, and β-globin. Median values are presented in Table S3 in Supplemental File 1.

**FIG 3:**
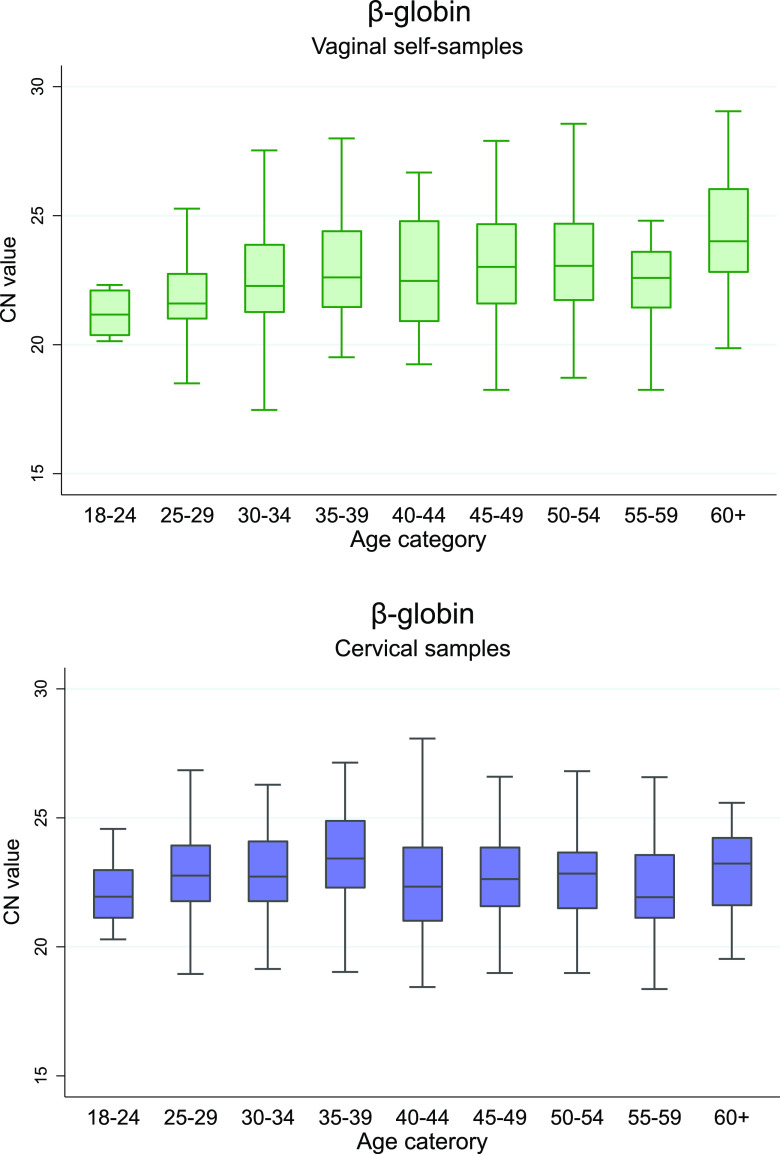
Boxplot of the RealTime β-globin cycle number by age group in vaginal (top) and cervical samples (bottom), by age group. The boxplot plots show median CN values, interquartile ranges, and extreme values (whiskers).

Median CN values were significantly lower in cervical samples indicating higher DNA concentration compared to vaginal samples collected with Evalyn Brush for HPV16 and other hrHPV, while in Qvintip samples only for other hrHPV (Fig. 2 and Table S3 in Supplemental File 1) (*P* < 0.05). A significant correlation was observed between vaginal and cervical CN values for HPV16, other hrHPV, and β-globin (*P* < 0.001), but not HPV18 (Fig. S1 in Supplemental File 1). Lower CN values were observed in cervical samples indicating higher DNA concentration in CIN2+ compared to <CIN2 for other hrHPV and β-globin, but not in vaginal samples (Table S4 and Fig. S2 in Supplemental File 1). In addition, we detected a significant increase in β-globin CN values by age in vaginal samples but not in cervical, representing a decrease in DNA concentration with age (Fig. 3 and Table S5 in Supplemental File 1). No association was observed between viral CN values and age (Table S5 in Supplemental File 1).

## DISCUSSION

In this study, we evaluated hrHPV DNA testing with the Abbott RT HPV assay on vaginal self-samples collected with two commercial devices, Evalyn Brush and Qvintip, with matched clinician-taken cervical samples within the VALHUDES framework.

Our findings showed lower clinical sensitivity on vaginal self-samples compared to cervical samples collected by a clinician. Because HPV tests have been validated to detect CIN2+ on cervical samples but not on other specimen types (14) an *a posteriori* exploratory cutoff optimization was carried out. After adjusting CN values to ≤35 on vaginal self-samples, the sensitivity for CIN2+ could be improved, but at the expense of a lowered specificity. One of the reasons for lower sensitivity on vaginal samples could be that samples were suspended at 20 mL of ThinPrep PreservCyt solution, representing an internationally recognized standard volume for clinician-collected cervical samples using ThinPrep. Cervical samples are taken by a gynecologist targeting the transformation zone, which is the predilection area for HPV infection and replication (19). On the other hand, vaginal samples are collected from the vagina where the concentration of HPV-infected cells is lower (20). It is therefore plausible to consider an analytically more sensitive cutoff or to explore smaller volumes of transport medium. Suspending the vaginal swab in 2 mL, for example, instead of 20 mL would be a 10-fold concentration increase and could generate positive signals around 3 cycles earlier (lower CN value). Such an increase in concentration could improve sensitivity and prevent cutoff optimization. Therefore, technical details such as the suspension volume of vaginal samples and cutoff values deserve further investigations and validations. Because Evalyn and Qvintip brushes were collected after using a Multi-collect swab, we could not exclude that the order of the vaginal sample collection might have influenced HPV detection; however, no effect of sample order was observed in previous studies (21, 22).

A previous meta-analysis failed to identify differences in the clinical accuracy of HPV testing of vaginal self-specimens collected with different devices, possibly lacking head-to-head comparisons (5). This VALHUDES study suggests that hrHPV testing to detect CIN2+ on samples collected with the Evalyn Brush was more accurate than with Qvintip. The recent PREDICTOR 5.1 trial assessed the clinical accuracy of the BD Onclarity HPV Assay on different self-sampling devices (dry flocked swab, Dacron swab, HerSwab, and Qvintip) (22). The vaginal samples were suspended in 8 mL PreservCyt solution, whereas VALHUDES used 20 mL. In addition, women were instructed to rotate Qvintip brushes five times in PREDICTOR 5.1 (personal communication Jack Cuzick) and once in VALHUDES. Despite the different sample collection processes and concentrations, both studies reported lower sensitivity values with Qvintip samples compared to Evalyn Brush in VALHUDES and flocked and Dacron swabs in PREDICTOR 5.1. Yet, cutoff optimization resulted in accuracy improvement in both trials (22). Furthermore, the only study to date with a direct comparison of Evalyn and Qvintip Brushes by Jentscke et al. suggested superior clinical performance of Evalyn Brush, although the power was limited due to the small sample size (21). Ørnskov et al. (23) evaluated the clinical performance of HPV testing using the Cobas HPV assay on cervical and vaginal samples collected with Evalyn Brush (vaginal samples were also suspended in 20 mL ThinPrep PreservCyt solution). In agreement with VALHUDES, similar sensitivity on vaginal Evalyn-Brush samples was observed compared to clinician-taken cervical samples (23). Another trial used Qvintip brushes suspended in 20 mL PreservCyt solution to evaluate different self-sampling strategies for non-responders in the Flemish cervical cancer screening program (24). Interestingly, our study confirms the report of the Flemish trial demonstrating a decrease in DNA concentration with age in vaginal samples. Lower cellularity in older women can be explained by reduced exfoliation of cervicovaginal epithelial cells (25) and therefore may result in lower DNA concentration in vaginal samples.

Two consecutive meta-analyses have reported similar sensitivity of PCR-based hrHPV assays on vaginal self-collected compared to cervical samples, whereas tests based on signal amplification were less sensitive on self-samples (5, 26).

HPV testing on self-samples for cervical cancer prevention has attracted attention from the scientific community and public health authorities over the last decade. Self-sampling has an immense potential to improve population coverage as it is a non-invasive, user-friendly, and cost-effective method to reach women not participating in the regular cervical cancer screening programs (11, 27, 28). Multiple studies have shown that offering a self-sampling device is more effective in reaching out to nonattendees than a routine invitation or reminder letter (5, 29). The 2020 WHO call to eliminate cervical cancer aimed to reach at least 70% coverage and the implementation of self-sampling strategies may contribute substantially to attaining this goal (30). Following these recommendations, several countries have initiated self-sampling within their organized HPV-based cervical cancer screening, including Australia, Denmark, the Netherlands, and United Kingdom (31–34).

The main strengths of our study were sample size empowered to verify accuracy hypotheses in a setting with a high prevalence of CIN2+, use of histologically defined outcome, sample processing for cervical specimens according to European guidelines for cervical cancer screening (18), and the use of an HPV DNA assay validated for cervical cancer screening on cervical samples (14). Moreover, our trial was conducted according to international STARD guidelines for the diagnostic test accuracy assessment (35). However, a colposcopy clinic, where VALHUDES was conducted is not representative of a screening setting where self-sampling will be mainly used. Nevertheless, our findings are in line with early results from the Dutch HPV-based screening program, including 30,808 vaginal also collected with Evalyn Brush and 456,207 clinician-collected cervical samples (33). The Evalyn Brush samples in the Dutch program were also placed in 20 mL PreservCyt solution. The average viral CN value which is inversely correlated with viral load was higher in vaginal samples than in cervical in our trial and the Dutch program. The Dutch report demonstrated good but some different performance of the Cobas 4800 HPV assay on the self-collected sample compared to the clinician-taken samples. Similarly, our study showed somewhat lower clinical sensitivity of the Abbott RT on vaginal samples, which was resolved by applying a higher CN threshold; a similar effect could be achieved by transferring the vaginal brush to a smaller volume. Alongside cutoff optimization, general workflow and sample handling procedures for vaginal self-samples may require standardized international consensus (36).

hrHPV testing with the Abbott RT assay on vaginal self-samples taken with the Evalyn Brush has similar accuracy to detect CIN2+ compared to testing on clinician-taken cervical samples. However, after cutoff optimization, similar relative sensitivity (self-taken versus clinician-taken samples) was also found for Qvintip specimens. Laboratory protocols and technical details such as dilution volume and cutoff values of HPV testing on self-samples warrant further investigation and optimization.

## MATERIALS AND METHODS

### Study design.

The VALHUDES concept (NCT03064087) follows a diagnostic test accuracy design and was established for validation of HPV assays on self-collected vaginal and urine samples as previously described (16). Briefly, a total of 523 women (median age, 40; interquartile range [IQR] 31 to 49) were recruited at five Belgian colposcopy centers (University Hospitals of Antwerp [UZA], Brussels [UZ Brussels], Ghent [UZ Ghent], Liège [CHU de Liège], and the General Regional Hospital Heilig Hart Tienen [RZ Tienen]) between December 2017 and January 2020. Women were referred to colposcopy due to previous cervical abnormality or HPV infection. Exclusion criteria were pregnancy, hysterectomy, incapability to understand and sign an informed consent, and refusal to participate in the study. Women were invited to participate in the study by phone call preceding the visit to the colposcopy service. All enrolled study participants signed an informed consent form and filled in a questionnaire. Ethical approval was obtained from the central Ethics Committee of the University Hospital of Antwerp/University of Antwerp (B300201733869) and the local Ethics Committees of all the centers involved.

Women who accepted participation in the trial received two Colli-Pee devices at their home address to collect two first-void urine samples the day before visiting the colposcopy clinic (17). Upon arrival at the colposcopy clinic, women were instructed to collect two vaginal self-samples, first with a Multi-Collect swab (Abbott GmbH, Wiesbaden, Germany), followed by a second self-sample with either Evalyn Brush (Rovers Medical Devices, Oss, The Netherlands) or Qvintip (Aprovix AB, Stockholm, Sweden). Women were instructed to insert Evalyn Brushes into the vaginal tract and rotate five times before removal (16). For Qvintip Brushes only one rotation was performed following the manufacturer´s instructions at the date of the study. In the current manuscript, we reported findings of Abbott RT testing only on vaginal samples collected with Evalyn Brush or Qvintip. Results of the Abbott RT test on urine samples are published elsewhere (17), whereas results of the Abbott Multi-Collect swab will be reported later.

Cervical samples were collected with a Cervex-Brush (Rovers Medical Devices, Oss, The Netherlands) by gynecologists shortly thereafter followed by colposcopy and biopsy specimen if indicated. Cervical specimens were placed in a vial containing 20 mL ThinPrep PreservCyt medium (Hologic, Inc., Bedford, MA, USA) in accordance with standard European guidelines for processing liquid-based cervical material (18). Dry vaginal brushes and cervical samples were stored at room temperature for a maximum of 6 days at the colposcopy clinic before dispatching to Algemeen Medisch Laboratorium (AML [Antwerp, Belgium]) for storage and further preprocessing. Upon arrival at AML, Evalyn and Qvintip brush heads were transferred into 20 mL ThinPrep PreservCyt vials. In the laboratory, the cervical and vaginal specimens were stored in a refrigerator at 4°C for a maximum of 72 days before HPV testing and further aliquoting into 1 mL aliquots. Thereafter, aliquots were frozen at −80°C at the AML biobank (Biobank, BB190002).

### HPV testing.

HPV testing on all samples was performed at AML with the Abbott RT test, which is the real-time PCR-based assay. All specimens (urine, cervical and vaginal) were tested on the same run by transferring 0.7 mL aliquot to an Abbott *m*2000 reaction vessel for automated HPV testing using the Abbott *m*2000 system following the manufacturer’s instructions (0.4 mL of input volume was processed by the system) (37). The remainder volume of each sample type of the ThinPrep vial was divided into 1 mL aliquots and stored at AML at −80°C (Biobank, BB190002) (16). The Abbott *m*2000 system performs DNA extraction, amplification, and interpretation of test results. The assay identifies and distinguishes HPV16, HPV18 from a pool of 12 other hrHPV types (HPV 31, 33, 35, 39, 45, 51, 52, 56, 58, 59, 66, and 68). The HPV target sequence for Abbott RT was in the conserved L1 region of the genome. Human β-globin was amplified as an internal control for specimen adequacy in a single reaction. Negative and positive controls were included in each run to assess run validity (37).

### Data analysis.

The Abbott RT assay on clinician-collected cervical samples was considered the comparator test, and the same test on vaginal self-samples as the index test, whereas colposcopy and histological examination of biopsy specimens were used as the reference standard. When colposcopy was satisfactory, did not reveal abnormal findings and no biopsy specimen was taken, the clinical outcome was classified as <CIN2. In all cases where a biopsy specimen was taken, the histopathology result was used as a standard outcome. In the case of multiple biopsy specimens, the histological outcome with the highest severity of disease was used. Samples with a cycle number (CN) of ≤32 as predefined by the manufacturer were considered HPV-positive. An exploratory cutoff optimization was performed to evaluate the impact of a higher CN threshold (CN ≤35) on the clinical sensitivity and specificity of the Abbott RT assay.

To evaluate the differences in sensitivity and specificity between the specimens, we used the McNemar test. Cohen’s Kappa was used to evaluate the concordance between the vaginal and cervical samples and was categorized as follows: 0.00 to 0.19 as poor, 0.20 to 0.39 as fair, 0.40 to 0.59 as moderate, 0.60 to 0.79 as good and 0.80 to 1.00 as excellent concordance (38, 39). Wilcoxon signed-rank and Mann-Whitney tests were performed to compare the differences in age and CN values for independent and matched comparison, respectively. Quantile regression was used to study the relations between CN values and age. Statistical analyses were performed using Stata 14.2 (College Station, TX, USA).
