# Supplementary material for: Clinical Performance of the RealTime High Risk HPV Assay on Self-Collected Vaginal Samples within the VALHUDES Framework
Source: Microbiol Spectr. 2022 Sep 1;10(5):e01631-22. doi: 10.1128/spectrum.01631-22 (PMC9602690; doi:10.1128/spectrum.01631-22)
Supplement: Supplemental file 1 — Supplemental material. Download spectrum.01631-22-s0001.pdf, PDF file, 0.4 MB [file spectrum.01631-22-s0001.pdf]

Supplementary Material

The clinical performance of the RealTi*me* High Risk HPV assay on self-collected vaginal samples within the VALHUDES framework

Excluded samples

- No urine samples were collected (n=4)
- No cervical samples were collected (n=2)
- No vaginal sample were collected (n=1)
- No Multi-Collect were collected (n=1)
- No colposcopy performed (n=1)
- Inadequate biopsy (1)
- Major protocol violations on urine samples (n=14)
- Invalid HPV test result on vaginal samples (n=8)
- Invalid HPV test result on cervical samples (n=5)

Supplementary Table S1**.** HPV prevalence and disease outcome by age group and total study population.

		Cervical hrHPV	Vaginal (E+Q) hrHPV CN≤32[a]	Vaginal (E+Q) hrHPV CN≤35[b]	Disease outcome		
Age category	Participants N (%)	Pos N (%)	Pos N (%)	Pos N (%)	<CIN2 N (%)	CIN2+ N (%)	CIN3+ N (%)
<30	96 (19.8)	65 (22.7)	64 (23.5)	71 (23.8)	73 (18.2)	23 (27.4	10 (23.3)
30-34	84 (17.3)	56 (19.6)	51 (18.8)	53 (17.8)	65 (16.2)	19 (22.6)	10 (23.3)
35-39	53 (10.9)	23 (8.0)	29 (10.7)	31 (10.4)	45 (11.2)	8 (9.5)	4 (9.3)
40-44	68 (14.0)	40 (14.0)	33 (12.1)	37 (12.4)	52 (12.9)	16 (19.1)	7 (16.3)
45-49	63 (13.0)	29 (10.1)	27 (9.9)	31 (10.4)	58 (14.4)	5 (6.0)	2 (4.7)
50-54	52 (10.7)	29 (10.1)	26 (9.6)	31 (10.4)	48 (11.9)	4 (4.8)	2 (4.7)
55-69	44 (9.1)	31 (10.8)	30 (11.0)	31 (10.4)	38 (9.5)	6 (7.1)	6 (14.0)
60+	26 (5.4)	13 (4.6)	12 (4.4)	13 (4.4)	23 (5.7)	3 (3.6)	2 (4.7)
Total	486 (100.0)	286 (100.0)	272 (100.0)	298 (100.0)	402 (100.0)	84 (100.0)	43 (100.0)

[a] Cut-off ≤ 32 cycle numbers (CN) as predefined by manufacturer.

[b] Exploratory cut-off ≤ 35 CNs for vaginal samples.

CIN, cervical intraepithelial neoplasia; E+Q, samples collected with Evalyn Brush (E) and Qvintip (Q) combined; N, number, Pos, positive.

Supplementary Table S2A. HrHPV concordance between vaginal (Evalyn + Qvintip) and cervical samples with exploratory cut off ≤ 35 cycle numbers.

	HPV type[a]	+/+	+/-	-/+	-/-	Concordance [%]	Kappa [95% CI]
**Total population (N=486)**	HrHPV	261	25	37	163	87.24	0.734 (0.673 - 0.796)
	HPV16	64	3	12	407	96.91	0.877 (0.816 - 0.938)
	HPV18	17	4	2	463	98.77	0.844 (0.721 - 0.967)
	Other hrHPV	210	26	41	209	86.21	0.725 (0.663 - 0.786)
	**HPV type[a]**	**+/+**	**+/-**	**-/+**	**-/-**	**Concordance [%]**	**Kappa [95% CI]**
**CIN3+ (N=43)**	HrHPV	39	3	0	1	93.02	0.377 (-0.155 - 0.909)
	HPV16	24	1	0	18	97.67	0.953 (0.861 - 1.000)
	HPV18	2	0	0	41	100	1.000 (1.000 - 1.000)
	Other hrHPV	20	3	3	17	86.05	0.720 (0.511 - 0.928)
	**HPV type**	**+/+**	**+/-**	**-/+**	**-/-**	**Concordance [%]**	**Kappa [95% CI]**
**CIN2+ (N=84)**	HrHPV	74	4	1	5	94.05	0.635 (0.343 - 0.928)
	HPV16	36	2	1	45	96.43	0.928 (0.847 - 1.000)
	HPV18	3	0	0	81	100.00	1.000 (1.000 - 1.000)
	Other hrHPV	48	4	5	27	89.29	0.771 (0.631 - 0.912)
	**HPV type**	**+/+**	**+/-**	**-/+**	**-/-**	**Concordance [%]**	**Kappa [95% CI]**
**<CIN2 (N=402)**	HrHPV	187	21	36	158	85.82	0.715 (0.647 - 0.784)
	HPV16	28	1	11	362	97.01	0.808 (0.702 - 0.913)
	HPV18	14	4	2	382	98.51	0.816 (0.672 - 0.960)
	Other hrHPV	162	22	36	182	85.57	0.711 (0.642 - 0.780)

Kappa concordance between the vaginal and cervical samples is presented as follows: 0.00 – 0.20 Poor; 0.21 – 0.40 Fair; 0.41 – 0.60 Moderate; 0.61 – 0.80 Good; 0.81 – 1.00 Excellent.

[a]Cut-off ≤ 32 cycle numbers (CN) as predefined by manufacturer for cervical samples and exploratory cut-off ≤ 35 for vaginal samples.

N, number; CI, 95% confidence interval; CIN, cervical intraepithelial neoplasia.

+/+ positive on vaginal and cervical samples, +/- positive only on cervical samples, -/+ positive only on vaginal samples, -/- negative on both sample types.

Supplementary Table S2B. HrHPV concordance between sample collected with Evalyn Brush and cervical samples with exploratory cut off ≤ 35 cycle numbers.

	HPV type[a]	+/+	+/-	-/+	-/-	Concordance [%]	Kappa [95% CI]
**Total population (N=228)**	HrHPV	128	12	10	78	90.35	0.797 (0.717 - 0.878)
	HPV16	38	2	3	185	97.81	0.925 (0.860 - 0.990)
	HPV18	12	4	1	211	97.81	0.816 (0.659 - 0.973)
	Other hrHPV	93	13	16	106	87.28	0.745 (0.658 - 0.832)
**CIN3+ (N=23)**	**HPV type[a]**	**+/+**	**+/-**	**-/+**	**-/-**	**Concordance [%]**	**Kappa [95% CI]**
	HrHPV	22	1	0	1	95.65	0.646 (0.012 - 1.000)
	HPV16	17	1	0	5	95.65	0.881 (0.654 - 1.000)
	HPV18	23	0	0	0	N/A	N/A
	Other hrHPV	7	1	2	13	86.96	0.721 (0.428 - 1.000)
**CIN2+ (N=47)**	**HPV type**	**+/+**	**+/-**	**-/+**	**-/-**	**Concordance [%]**	**Kappa [95% CI]**
	HrHPV	43	1	0	3	97.87	0.846 (0.551 - 1.000)
	HPV16	26	2	0	19	95.74	0.913 (0.796 - 1.000)
	HPV18	1	0	0	46	100.00	1.000 (1.000 - 1.000)
	Other hrHPV	23	1	3	20	91.49	0.829 (0.670 - 0.989)
**<CIN2 (N=181)**	**HPV type**	**+/+**	**+/-**	**-/+**	**-/-**	**Concordance [%]**	**Kappa [95% CI]**
	HrHPV	85	11	10	75	88.40	0.767 (0.674 - 0.861)
	HPV16	12	0	3	166	98.34	0.880 (0.746 - 1.000)
	HPV18	11	4	1	165	97.24	0.800 (0.630 - 0.970)
	Other hrHPV	70	12	13	86	86.19	0.722 (0.620 - 0.823)

Kappa concordance between the vaginal and cervical samples is presented as follows: 0.00 – 0.20 Poor; 0.21 – 0.40 Fair; 0.41 – 0.60 Moderate; 0.61 – 0.80 Good; 0.81 – 1.00 Excellent.

[a]Cut-off ≤ 32 cycle numbers (CN) as predefined by manufacturer for cervical samples and exploratory cut-off ≤ 35 for vaginal samples.

N, number; CI, 95% confidence interval; CIN, cervical intraepithelial neoplasia; N/A, not available.

+/+ positive on vaginal and cervical samples, +/- positive only on cervical samples, -/+ positive only on vaginal samples, -/- negative on both sample types.

Supplementary Table S2C. HrHPV concordance between sample collected with Qvintip and cervical samples with exploratory cut off ≤ 35 cycle numbers.

	HPV type[a]	+/+	+/-	-/+	-/-	Concordance [%]	Kappa [95% CI]
**Total population (N=258)**	HrHPV	133	13	27	85	84.50	0.680 (0.589 - 0.770)
	HPV16	26	1	9	222	96.12	0.817 (0.708 - 0.927)
	HPV18	5	0	1	252	99.61	0.907 (0.726 - 1.000)
	Other hrHPV	117	13	25	103	85.27	0.705 (0.619 - 0.791)
	**HPV type[a]**	**+/+**	**+/-**	**-/+**	**-/-**	**Concordance [%]**	**Kappa [95% CI]**
**CIN3+ (N=20)**	HrHPV	18	2	0	0	90.00	N/A
	HPV16	7	0	0	13	100	1.000 (1.000 - 1.000)
	HPV18	2	0	0	18	100	1.000 (1.000 - 1.000)
	Other hrHPV	13	2	1	4	85.00	0.625 (0.243 - 1.000)
	**HPV type**	**+/+**	**+/-**	**-/+**	**-/-**	**Concordance [%]**	**Kappa [95% CI]**
**CIN2+ (N=37)**	HrHPV	31	3	1	2	89.19	0.444 (-0.006 - 0.894)
	HPV16	10	0	1	26	97.30	0.934 (0.805 - 1.000
	HPV18	2	0	0	35	100.00	1.000 (1.000 - 1.000)
	Other hrHPV	25	3	2	7	86.49	0.646 (0.363 - 0.929)
	**HPV type**	**+/+**	**+/-**	**-/+**	**-/-**	**Concordance [%]**	**Kappa [95% CI]**
**<CIN2 (N=221)**	HrHPV	102	10	26	83	83.71	0.674 (0.577 - 0.770)
	HPV16	16	1	8	196	95.93	0.759 (0.608 - 0.909)
	HPV18	3	0	1	217	99.55	0.855 (0.574 - 1.000)
	Other hrHPV	92	10	23	96	85.07	0.702 (0.609 - 0.795

Kappa concordance between the vaginal and cervical samples is presented as follows: 0.00 – 0.20 Poor; 0.21 – 0.40 Fair; 0.41 – 0.60 Moderate; 0.61 – 0.80 Good; 0.81 – 1.00 Excellent.

[a]Cut-off ≤ 32 cycle numbers (CN) as predefined by manufacturer for cervical samples and exploratory cut-off ≤ 35 for vaginal samples.

N, number; CI, 95% confidence interval; CIN, cervical intraepithelial neoplasia; N/A, not available.

+/+ positive on vaginal and cervical samples, +/- positive only on cervical samples, -/+ positive only on vaginal samples, -/- negative on both sample types.

Supplementary Table S3A. Comparison of median CN values between cervical and vaginal samples.

	N	Median CN cervical [IQR]	Median CN vaginal (E+Q) [IQR]	p-value paired t-test
**HPV16**	63	20.3 [18.2-23.2]	22.0 [20.4-23.9]	0.0006
**HPV18**	16	22.4 [19.7-25.7]	22.9 [18.1-25.0]	0.3254
**Other hrHPV**	196	21.9 [18.8-24.6]	22.7 [18.9-26.1]	0.0004
**Beta-globin**	486	22.6 [21.5- 24.1]	22.4 [21.3-23.9]	0.4958

Supplementary Table S3B. Comparison of median CN values between cervical and vaginal samples collected with Evalyn Brush

	N	Median CN cervical [IQR]	Median CN Evalyn [IQR]	p-value paired t-test
**HPV16**	38	20.5 [18.3-23.6]	22.9 [20.8-27.1]	0.0038
**HPV18**	12	23.9 [19.7-26.5]	23.2 [18.6-26.2]	0.3882
**Other hrHPV**	86	22.4 [19.6-25.5]	24.2 [20.0-26.6]	0.0074
**Beta-globin**	228	22.6 [21.6- 24.1]	22.6 [21.2-24.4]	0.8852

Supplementary Table S3C. Comparison of median CN values between cervical and vaginal samples collected with Qvintip.

	N	Median CN cervical [IQR]	Median CN Qvintip [IQR]	p-value paired t-test
**HPV16**	25	20.1 [18.0-21.9]	21.4 [19.4-22.6]	0.1462
**HPV18**	4	21.0 [18.5-22.7]	20.7 [15.9-23.1]	0.4652
**Other hrHPV**	110	21.6 [18.3- 23.9]	22.3 [18.3- 25.9]	0.0208
**Beta-globin**	258	22.6 [21.5- 24.1]	22.2 [21.3-23.7]	0.2577

Cut-off ≤ 32 cycle numbers (CN) as predefined by manufacturer.
IQR, interquartile range (25-75%); E+Q, samples collected with Evalyn Brush (E) and Qvintip (Q) combined; N, number.
Mann-Whitney test was used to compare differences in median CN values of matched cervical and vaginal samples.

Supplementary Table S4A. Comparison of median CN values for cervical samples between CIN2+ and <CIN2.

	N	Median CN <CIN2 [IQR]	Median CN CIN2+ [IQR]	p-value paired t-test
**HPV16**	29/38	21.7 [18.2-25.0]	20.2 [18.3- 23.2]	0.4072
**HPV18**	18/3	22.4 [19.19-26.8]	23.6 [16.7-24.8]	0.6153
**Other hrHPV**	184/52	22.7 [20.0-26.1]	21.2 [18.7- 24.6]	0.0436
**Beta-globin**	402/84	22.8 [21.5- 24.2]	22.4 [21.4-23.4]	0.0236

Supplementary Table 4B. Comparison of median CN values for vaginal samples between CIN2+ and <CIN2.

	N	Median CN <CIN2 [IQR]	Median CN CIN2+ [IQR]	p-value paired t-test
**HPV16**	34/37	21.8 [20.2-26.4]	22.7 [21.3-23.9]	0.7126
**HPV18**	15/3	22.9 [17.9-26.6]	23.2 [23.0-23.6]	0.8590
**Other hrHPV**	173/48	23.6 [19.7-26.9]	22.2 [18.7-25.4]	0.2145
**Beta-globin**	402/84	22.5 [21.3-24.2]	22.2 [21.1-23.7]	0.1551

[a] number of HPV positive samples in women with <CIN2 versus CIN2+

Cut-off ≤ 32 cycle numbers (CN) as predefined by manufacturer.

IQR, interquartile range (25-75%); CIN, cervical intraepithelial neoplasia; E+Q, samples collected with Evalyn Brush (E) and Qvintip (Q) combined; N, number. Wilcoxon signed-rank test was used to compare differences in median CN values.

Supplementary Table S5A. Association between age and CN values using quantile regression analysis in cervical samples.

Age	Coef.	p-value	[95% Conf	Interval]
HPV16	0.058	0.586	-0.151	0.267
HPV18	-0.154	0.389	-0.507	-0.507
Other hrHPV	-0.057	0.416	-0.197	0.081
Beta-globin	-0.524	0.236	-1.393	0.344

Supplementary Table S5B. Association between age and CN values using quantile regression analysis in vaginal self-samples (Evalyn Brush +Qvintip).

Age	Coef.	p-value	[95% Conf	Interval]
HPV16	-0.130	0.195	-0.327	0.067
HPV18	0.037	0.851	-0.355	0.431
Other hrHPV	-0.122	0.080	-0.259	0.014
Beta-globin	1.282	<0.001	0.563	2.000

Coef, quantile regression coefficient.

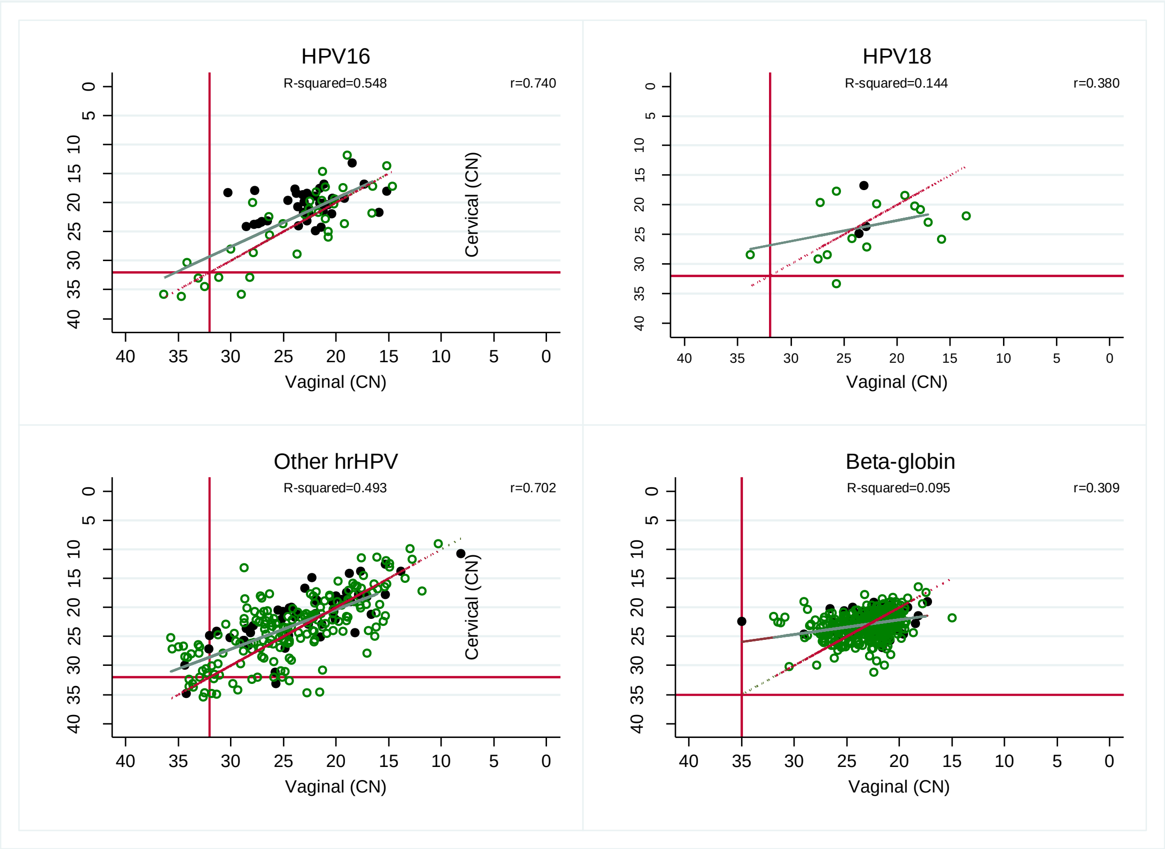

Supplementary Figure S1A-D. Scatter plots presenting correlation in cycle numbers (CN) values between cervical and vaginal self-samples for HPV16, HPV18, other hrHPV and human β-globin. Samples with CNs equal to zero are samples with undetectable HPV or human β-globin and therefore are excluded from the scatter plot. Red vertical and horizontal lines represent the manufacturer defined CN cut-offs.

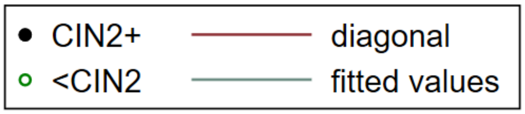

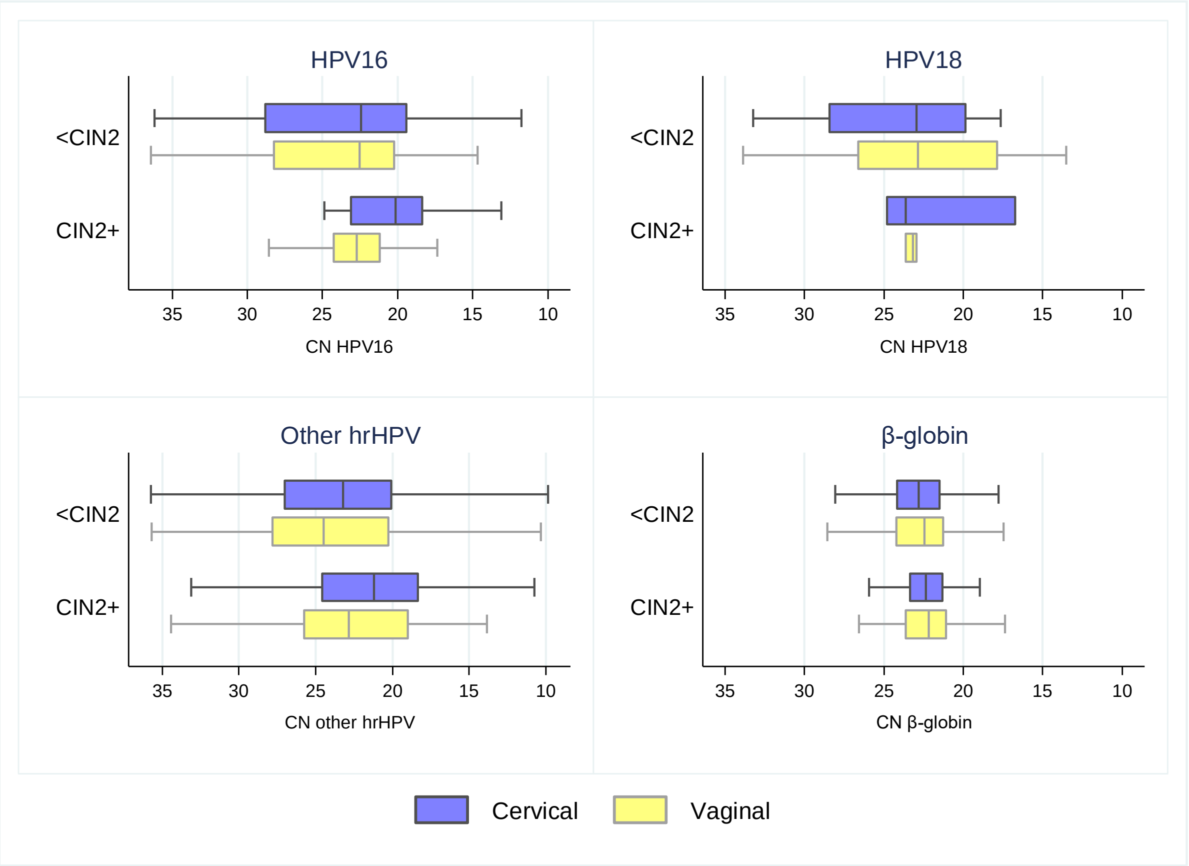

Supplementary Figure S2. RealTi*me* High Risk HPV cycle number (CN) values on cervical and vaginal samples with CIN2+ compared to <CIN2. Boxplots indicate median CN-values, interquartile ranges and extreme values (whiskers) for HPV16, 18, other hrHPV and β-globin. Median values are presented in Supplementary Table S4.
